# Digital interaction and active aging: the impact of social media use on physical activity behavior mediated by social capital and self-efficacy

**DOI:** 10.3389/fpsyg.2025.1580936

**Published:** 2025-06-20

**Authors:** Jian Liu, Chaoxin Wang, Zhanpeng Meng, Chuanwen Yu

**Affiliations:** ^1^College of Physical Education and Health, Heze University, Heze, China; ^2^College of Physical Education, Shandong Normal University, Jinan, China

**Keywords:** social media, social capital, self-efficacy, physical activity behavior, older adults

## Abstract

**Background:**

Social media use has been linked to higher physical activity levels in older adults, but the mechanisms underlying this connection are not yet well understood. Emerging evidence suggests that social capital may act as a mediator, though little research has explored whether specific dimensions of social capital and self-efficacy mediate this relationship.

**Objective:**

This study investigates how social media usage influences older adults’ physical activity behavior, focusing on the mediating roles of social capital—structural, bonding, and bridging—and self-efficacy. The goal is to provide a foundation for strategies to promote physical activity in this population.

**Methods:**

A cross-sectional survey was conducted between December 2023 and April 2024 involving 519 social media users (275 male and 244 female). Data were gathered using validated scales for social media usage intensity, physical activity levels (PARS-3), social capital, and self-efficacy. Structural equation modeling (SEM) was applied to examine the relationships between these variables and the mediating effects at play.

**Results:**

Older adults who engaged more actively with social media exhibited higher physical activity behavior (*β* = 0.179, *p* < 0.05). Social capital, across its three dimensions—structural (*β* = 0.254), bonding (*β* = 0.294), and bridging (*β* = 0.237)—significantly mediated the link between social media usage and physical activity (all *p* < 0.05). Additionally, self-efficacy was a critical, independent mediator (*β* = 0.242, *p* < 0.05). A chain-mediating effect involving social capital dimensions and self-efficacy further strengthened this relationship (*p* < 0.05).

**Conclusion:**

Social media use fosters physical activity in older adults by building social capital, mainly structural, bonding, and bridging types, and boosting self-efficacy. Enhancing the social media environment, developing social capital, and supporting self-efficacy are vital strategies for promoting physical activity in this group. The cross-sectional design of this study is a limitation, and future longitudinal research is needed to understand the causal relationships better.

## Introduction

The rapid acceleration of global aging has highlighted the urgent need to promote healthy aging (Chen et al., 2022b). Research consistently shows that regular physical activity behavior enhances older adults’ physical functions, mental well-being, and social adaptability (Xiong et al., 2021; Liu et al., 2024). However, due to declines in physical abilities and shrinking social networks, older adults often engage in less physical activity (Sun et al., 2024). Therefore, developing effective strategies to encourage this population’s physical activity is critical.

Recent advancements in digital technology have created new opportunities to address these challenges. Social media, in particular, has allowed older adults to overcome barriers related to geography and physical limitations, thereby revitalizing their social networks (Liu and Zhao, 2023). It also serves as a platform for accessing health-related information, sharing experiences, and receiving social support (Tseng et al., 2022). While evidence suggests that social media positively influences health behaviors in older adults (Asiamah et al., 2021), research on its impact specifically on physical activity behavior remains limited (López-Carril et al., 2020; Wang and Xu, 2024).

The role of social media in improving life satisfaction (Zhao, 2021) and facilitating psychosocial outcomes such as social support and social capital (Liu and Yeo, 2022). While much of this research focuses on adolescents (Schemer et al., 2021), college students (Roberts and David, 2020), and general adult populations (Chen and Li, 2017), studies on older adults are still in the early stages. Social media use can enhance self-efficacy and social support, key factors in promoting physical activity (Steinhoff and Reiner, 2024). By enabling the formation of social support networks and providing access to health-related resources, social media has significant potential to encourage physical activity among older adults (Lin and Kishore, 2021; Yi et al., 2024). Moreover, the self-efficacy gained through online interactions may play a pivotal role in shaping exercise behavior among older adults (Sen et al., 2022; Long et al., 2024).

The theoretical basis for this model is grounded in Social Cognitive Theory (Bandura, 1989) and the Socioecological Model (Bronfenbrenner, 1979). Social Cognitive Theory emphasizes the role of observational learning, social support, and self-regulation in health behavior. At the same time, the Socioecological Model highlights the influence of multiple levels of social environments, including digital platforms, on individual behaviors. Social media serves as a tool for strengthening social networks (structural, bonding, and bridging social capital), providing emotional support and behavioral resources (Huang et al., 2024), and enhancing the motivation to engage in physical activity behavior. Additionally, through its interactive nature, social media boosts self-efficacy by reinforcing confidence in one’s ability to participate in physical activity.

This study introduces several key innovations. First, it is the first to construct a chain mediation model that simultaneously incorporates three dimensions of social capital—structural, bonding, and bridging—along with self-efficacy to explore how social media use influences physical activity behavior among older adults. Unlike previous studies that often emphasize cognitive or linking social capital (Solano, 2025; Rahe et al., 2025; Zhang et al., 2025), this research strategically focuses on the three dimensions most directly related to health behaviors, thereby enhancing the explanatory power and practical relevance of the model. Second, by differentiating the roles of bonding, bridging, and structural capital, the study clarifies the specific mechanisms through which social media platforms, such as WeChat, foster distinct social connections in later life, providing a more nuanced understanding of digital engagement. Third, the research reflects a localized innovation by embedding the analysis within the Chinese sociocultural context, where family-oriented networks and the widespread use of WeChat shape online interaction patterns. This approach highlights the unique function of WeChat in strengthening bonding capital and promoting physical activity among older adults. Finally, the study extends the theoretical framework of social capital and health behavior by introducing a complex, multi-level mediation structure, providing a novel direction for future empirical and intervention research.

## Literature review

**Figure 1:**
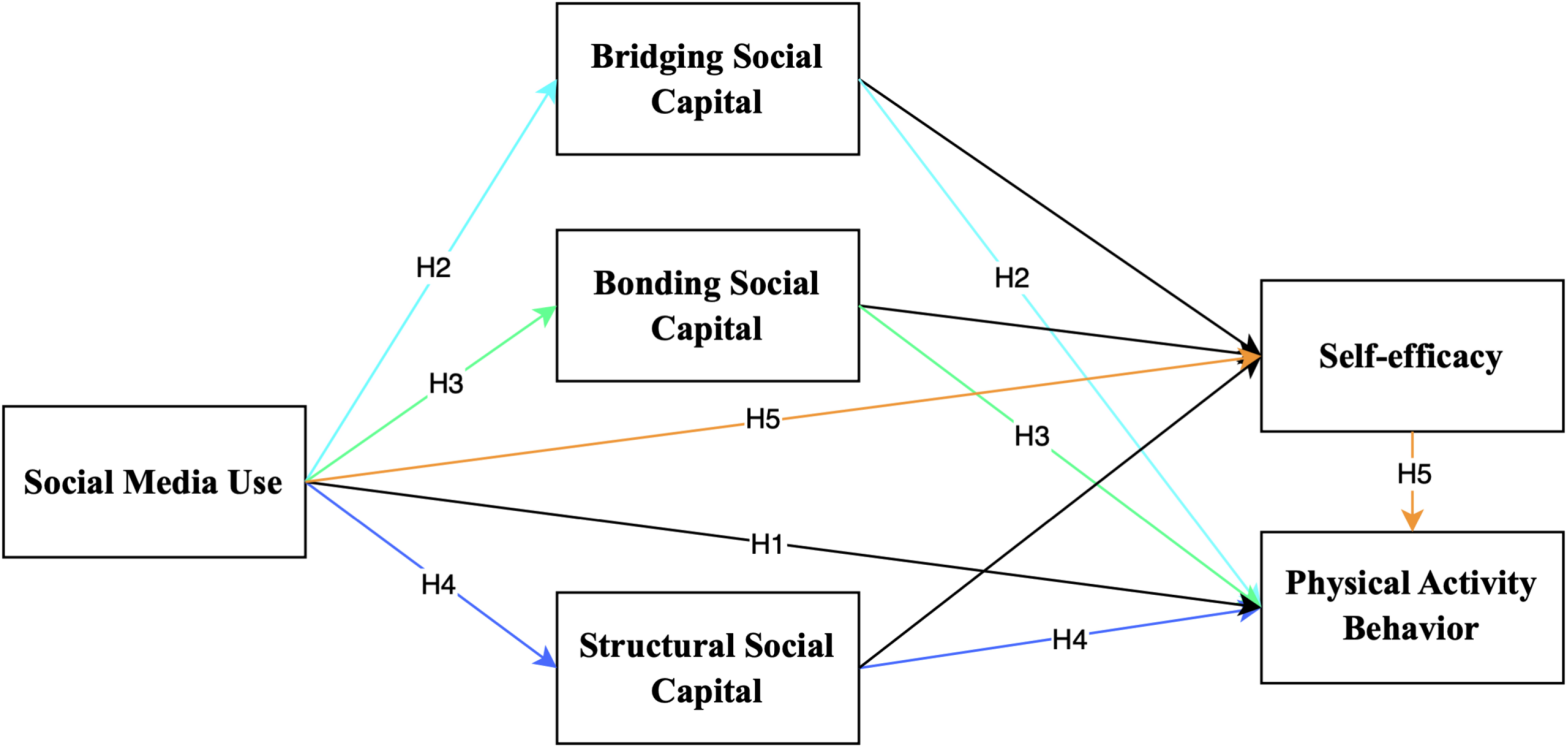
Conceptual framework illustrating the hypothesized relationships among social media use, social capital dimensions, self-efficacy, and physical activity behavior in older adults.

To further elaborate on the theoretical foundations and clarify the research gap identified in the introduction, this section reviews prior studies on social media use, social capital, and physical activity among older adults, setting the stage for the study’s innovative framework. Regular social media use has been shown to help individuals build social capital and enhance their self-efficacy, which contributes to consistent exercise habits (Szwedo et al., 2024). The effects of social media on physical activity are often mediated by different dimensions of social capital, including bridging, bonding, and structural types (Gao et al., 2025). The conceptual framework is shown in Figure 1.

### Social media use and physical activity behavior

Social media platforms enable users to share information, interact with others, and access content, which has helped overcome the limitations of traditional media (Jitsaeng et al., 2024). The rapid growth of social media has aligned with advancements in Web 2.0 technologies, facilitating greater communication and information dissemination (Guaya et al., 2025). Notably, social media use among older adults has significantly increased (Dahlberg et al., 2022), especially in China, where it has become a vital tool for information acquisition and socializing (Celik, 2023; Wei and Gao, 2017). Several studies have explored how digital tools can support physical activity behavior among older adults. For instance, Jansons et al. (2022) found that exercise programs delivered through the Amazon Echo Show effectively encourage older adults to be more active. Similarly, Ho and Merchant (2022) demonstrated the effectiveness of remote guidance in managing exercise routines during the COVID-19 pandemic.

Additionally, studies have highlighted how apps like Social Bike and live-streamed exercise sessions via Facebook have improved physical activity behavior outcomes for older adults (Arlati et al., 2019; Chang et al., 2021). However, while these studies underscore the potential of social media to encourage physical activity, they fail to examine how social media use directly influences elderly exercise behavior, particularly through the lens of social capital and self-efficacy. Therefore, this study hypothesizes:

*H1: Social Media Use is positively associated with Physical activity behavior among older adults.*

### The mediation role of social capital and self-efficacy

The rise of digital media has transformed traditional communication patterns, replacing face-to-face interactions with online connections. Social media is a powerful platform for building social capital—the resources derived from social networks (Tuan et al., 2024). Unlike traditional social capital, which is grounded in physical interactions, digital social capital enables broader, more diverse connections, enhancing bonding and bridging social capital (Perez Fernandez et al., 2024). Structural social capital is the underlying framework that shapes these interactions (Nahapiet and Ghoshal, 1998). Social media has been shown to expand these forms of capital by lowering the costs of maintaining relationships, enabling the development of both strong ties within close-knit groups (bonding) and weak ties across different groups (bridging), both of which can promote physical activity (Kahai and Lei, 2019; Sachan et al., 2025). Social capital positively influences online and offline physical activity participation (Fan et al., 2023; Fu et al., 2018). Social capital facilitates the spread of health-related information, encouraging greater participation in exercise (Reid et al., 2016).

Additionally, increasing access to social media has expanded opportunities for exercise engagement among older adults (Zhong et al., 2022). These findings suggest that social media is pivotal in helping older adults develop the social networks and support systems necessary for sustained physical activity. Therefore, social media helps older adults expand their social networks, lengthen social capital, and serve as a key factor in increasing their engagement in physical activity behavior. The following hypotheses are proposed:

*H2: Bridging social capital mediates the relationship between social media use and physical activity behavior among older adults.*

*H3: Bonding social capital mediates the relationship between social media use and physical activity behavior among older adults.*

*H4: Structural social capital mediates the relationship between social media use and physical activity behavior among older adults.*

### Social capital, self-efficacy, and social media use

Self-efficacy—the belief in one’s ability to succeed in specific tasks—has been identified as a crucial determinant of physical activity in older adults (Fu et al., 2023). Research suggests that higher self-efficacy is associated with greater participation in moderate and high-intensity physical activities (Yu and Song, 2022). By providing access to health-related content and online communities, social media can enhance self-efficacy by offering emotional support and reinforcing positive health behaviors (Godfrey and Johnson, 2009; Guan, 2024). Studies have shown that older adults who engage with social networking sites exhibit higher levels of self-efficacy in physical activities, increasing their likelihood of participating in exercise (Chen et al., 2022a). Based on this, the following hypotheses are proposed:

*H5: Self-efficacy significantly mediates the relationship between social media use and older adults’ physical activity behavior.*

The interaction between social capital and self-efficacy is also crucial. Social capital enhances self-efficacy by providing resources such as emotional support, social recognition, and encouragement, strengthening individuals’ confidence in their ability to engage in physical activity (Kim et al., 2021; Patterson et al., 2022). The accumulation of structural and bridging social capital through social media can improve self-efficacy, as seen in studies that link Facebook-based exercise programs to better adherence and motivation for physical activity (Chang et al., 2021). Thus, this study incorporates both social capital and self-efficacy to propose the following hypotheses:

*H6: Social media use influences older adults' physical activity behavior through the sequential mediating roles of structural social capital and self-efficacy.*

*H7: Social media use influences older adults' physical activity behavior through the sequential mediating roles of bonding social capital and self-efficacy.*

*H8: Social media use influences older adults' physical activity behavior through the sequential mediating roles of bridging social capital and self-efficacy.*

## Methods

### Sample and procedure

This study investigates the impact of social media use, social capital, and self-efficacy on physical activity behavior among older adults. Shandong Province was selected as the research site due to its large population, high aging rate (21.5%, exceeding the national average of 18.8%), and relatively advanced economic and digital infrastructure. Participants were recruited from urban and rural communities across multiple regions of Shandong Province between December 1, 2023, and April 1, 2024. To enhance sample representativeness, a multi-stage stratified random sampling approach was employed. In the first stage, districts and counties were stratified by urban–rural classification and key socioeconomic indicators (e.g., average household income and educational attainment). In the second stage, specific communities were randomly selected within each stratum in collaboration with local civil affairs departments and community committees. Within the selected communities, community workers compiled rosters of eligible older adults. From these lists, participants were systematically sampled (e.g., every third name) and invited to participate. To ensure diversity across demographic variables, quotas were applied for age groups (60–69, 70–79, 80+), gender, and self-reported digital literacy levels. Participants were eligible to participate if they met the following criteria: (a) Aged 60 years or older; (b) In generally good physical health, as self-reported or verified by local health service records; (c) Capable of completing the questionnaire independently or with minimal assistance; (d) Reported at least occasional use of social media platforms (e.g., WeChat, QQ, Douyin), without restriction to a specific platform.

Technological proficiency may influence willingness and ability to participate, so measures were taken to reduce selection bias. Specifically, trained investigators conducted face-to-face interviews with older adults with limited formal education, low digital literacy, or difficulty reading. During these sessions, investigators read the questions aloud, explained items when necessary, and recorded responses verbatim, ensuring that individuals with lower levels of digital engagement were adequately represented. A total of 630 questionnaires were distributed. After eliminating incomplete or invalid responses, 519 valid questionnaires were obtained, yielding an effective response rate of 82.4%. The final sample encompassed a broad range of demographic characteristics (Table 1), enhancing the findings’ generalizability and robustness.

**Table 1:** Demographic characteristics of the participants.

Variables	Options	*N*	Percent (%)	Total
Gender	Male	275	53.00	519
	Female	244	47.00	
Age	60 ~ 69 years old	318	61.30	519
	70 ~ 79 years old	186	35.80	
	Over 80 years old	15	2.90	
Place of residence	Rural areas	240	46.20	519
	Urban areas	279	53.80	
Education	No formal education	34	6.60	519
	Primary school	154	29.70	
	Junior high school	162	31.20	
	Senior high school(including vocational school and technical school)	67	12.90	
	College or above (including associate degree, bachelor’s degree, and postgraduate degree)	102	19.70	
Monthly income	Less than 1,000$	95	18.30	519
	1,001–1,500$	122	23.50	
	1,501–2000$	138	26.60	
	More than 2000$	164	31.60	

### Measures

The measurement scales used in this study were adapted and validated for the Chinese older adult population. Initially developed by Sánchez-Arrieta et al. (2021), the social media usage intensity scale was modified to reflect the context of older adults in China. This adaptation process involved forward and back translation to ensure clarity and cultural relevance. The scale was validated through expert review and pilot testing, which confirmed its suitability for the target population. The final version of the scale includes five items, assessing older adults’ social media engagement and dependency in daily life, with responses recorded on a five-point Likert scale from 1 (strongly disagree) to 5 (strongly agree). This study’s scale demonstrated strong reliability, with a Cronbach’s alpha of 0.902. We used the Physical Activity Rating Scale (PARS-3) to measure physical activity behavior, as revised by Xiong et al. (2023). This scale evaluates physical activity across three dimensions: exercise intensity, session duration, and frequency, each rated on a five-point scale. The two-week test–retest reliability was 0.85, and the internal consistency coefficient was 0.92, indicating strong reliability. Social capital was assessed through three dimensions: structural social capital, bonding social capital, and bridging social capital, using a five-point Likert scale adapted from Williams (2006) Internet Social Capital Scale. The adapted scale was translated and validated for the Chinese context. Self-efficacy was measured using the General Self-Efficacy Scale (GSES) developed by Kahai and Lei (2019), consisting of 10 items rated on a five-point Likert scale, with higher scores indicating greater self-efficacy. The GSES demonstrated excellent reliability in this study, with a Cronbach’s alpha 0.950.

### Data analysis

Data analysis was conducted using SPSS 22.0. Harman’s single-factor test was employed to assess potential standard method bias, revealing six factors with eigenvalues greater than 1, which together explained a cumulative variance of 69.62%. The first factor accounted for 20.84% of the variance, well below the 40% threshold proposed by Podsakoff et al. (2003), indicating that common method bias was not a significant concern in this study. Although other methods for testing common method bias, such as the marker variable approach, exist, they were not employed here due to Harman’s test’s robustness and consistency with prior research findings.

Following the assessment of method bias, model fit was evaluated. The model demonstrated a strong fit, with CMIN/DF = 1.820 (<3), GFI = 0.914, AGFI = 0.899, NFI = 0.929, IFI = 0.921, TLI = 0.963, CFI = 0.969, and RMSEA = 0.040 (90% CI: 0.035–0.044). These values indicate a good fit for the data, meeting the criteria proposed by Kim (2005) and Hu and Bentler (1999).

Convergent validity was assessed to establish the psychometric soundness of the model further. The AVE values ranged from 0.603 to 0.651, and the CR values ranged from 0.864 to 0.951, which exceeded the recommended thresholds (Fornell and Larcker, 1981). Discriminant validity was evaluated using Fornell and Larcker’s (1981) method. As shown in Table 2, the square root of the AVE for each construct exceeded its correlations with other constructs, confirming that the scales effectively distinguished between different theoretical concepts. For example, the square root of the AVE for social media usage (0.78) was substantially greater than its correlation with physical activity behavior (*r* = 0.32), demonstrating successful construct differentiation.

**Table 2:** Pearson correlation analysis and AVE square root values.

Variable	SMU	BRI	STR	BON	SE	PAB
SMU	**0.807**					
BRI	0.438	**0.788**				
STR	0.498	0.192	**0.777**			
BON	0.461	0.016	0.344	**0.784**		
SE	0.634	0.361	0.529	0.594	**0.813**	
PAB	0.241	0.197	0.208	0.324	0.470	**0.757**

SMU, social media use; BRI, bridging social capital; STR, structural social capital; BON, bonding social capital; SE, self-efficacy; PAB, physical activity behavior. The bold value is the square root of AVE.

## Results

**Figure 2:**
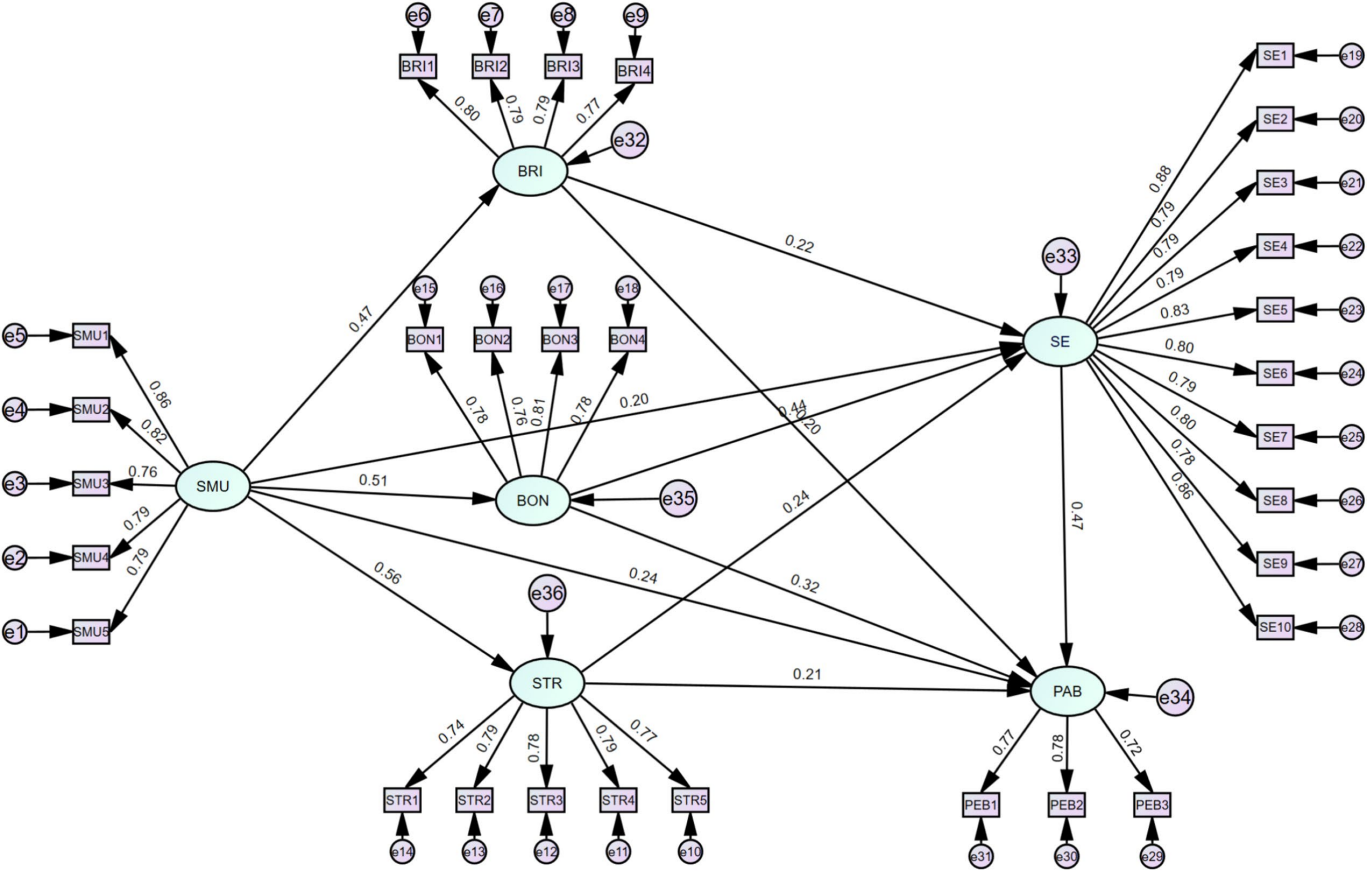
Structural equation model results showing the standardized path coefficients among social media use, social capital, self-efficacy, and physical activity behavior.

This study investigates the relationships between six latent variables: social media use, bridging social capital, bonding social capital, structural social capital, self-efficacy, and physical activity behavior. Specifically, social media use is treated as an exogenous latent variable. In contrast, the three dimensions of social capital (bridging, bonding, and structural), self-efficacy, and physical activity behavior are modeled as endogenous latent variables. A total of 31 observed variables were included in the analysis. Based on the proposed hypotheses, an SEM was constructed to examine the relationships between these variables and to explore how social media use influences physical activity behavior among older adults. The model structure is illustrated in Figure 2. Data analysis was conducted using AMOS 29.0 software, and the resulting fit indices are as follows: CMIN/DF = 1.820, GFI = 0.914, AGFI = 0.899, NFI = 0.929, IFI = 0.921, TLI = 0.963, CFI = 0.969, and RMSEA = 0.040, 90% CI [0.035–0.044]. All indices meet the recommended thresholds, indicating that the model adequately fits the data and demonstrates strong explanatory power regarding the impact of social media use on physical activity behavior among older adults. Furthermore, Table 3 presents the measurement model results, showing that all latent variables—social media use, bonding social capital, bridging social capital, structural social capital, and self-efficacy—passed the significance tests. The *p*-values for the path coefficients linking these latent variables to their respective observed variables are below 0.01, confirming the validity of the observed variables as indicators of the latent constructs.

**Table 3:** Results of SEM path relationship test on factors influencing physical activity behavior of the elderly.

Path	Estimate	S. E.	C. R.	*p*
BRI ← SMU	0.473	0.05	9.509	***
BON ← SMU	0.511	0.049	10.153	***
STR ← SMU	0.557	0.048	11.082	***
SE ← SMU	0.203	0.056	4.182	***
SE ← BRI	0.219	0.043	5.911	***
SE ← BRI	0.439	0.049	10.532	***
SE ← STR	0.245	0.049	6.147	***
PAB ← BRI	0.197	0.013	2.893	0.004
PAB ← BON	0.324	0.017	3.695	***
PAB ← STR	0.208	0.015	2.862	0.004
H1: PAB ← SMU	0.241	0.016	2.820	0.005
PAB ← SE	0.470	0.018	4.232	***

***p < 0.001.

As shown in Table 3, the path analysis supported all hypothesized relationships. Specifically, social media usage directly affected physical activity among older adults (*β* = 0.241, *p* = 0.005), confirming H1. It also significantly enhanced all three dimensions of social capital—bridging (*β* = 0.473), bonding (*β* = 0.511), and structural (*β* = 0.557)—as well as self-efficacy (*β* = 0.203), all at *p* < 0.001.

In turn, the three forms of social capital positively influenced self-efficacy (*β* = 0.219 for bridging, 0.439 for bonding, and 0.245 for structural), and both bridging and structural social capital were directly associated with higher physical activity (*β* = 0.197 and 0.208, respectively; *p* = 0.004). Finally, self-efficacy emerged as a strong predictor of physical activity (*β* = 0.470, *p* < 0.001).

Before testing the mediation paths, we verified the existence of multicollinearity among the mediators—structural social capital, bonding social capital, and bridging social capital. The results indicated that all mediators’ variance inflation factors (VIFs) were significantly below the threshold of 5, suggesting that multicollinearity is not a concern (Hair Jr et al., 2010). To further explore the mediation mechanisms, this study employed the Bootstrap method with 5,000 resamples to assess multiple mediation effects. Confidence intervals that do not contain zero were used as indicators of statistical significance. As shown in Table 4, structural social capital (total effect = 0.254, indirect effect = 0.074), bonding social capital (total effect = 0.294, indirect effect = 0.114), and bridging social capital (total effect = 0.237, indirect effect = 0.057) all partially mediate the relationship between social media use and physical activity behavior among older adults. These findings fully support Hypotheses H2, H3, and H4. When self-efficacy is considered a mediator, the total effect is 0.242 (direct effect = 0.179, indirect effect = 0.063), indicating partial mediation and fully supporting Hypothesis H5.

**Table 4:** Mediation analysis of the effects of social capital dimensions and self-efficacy on the relationship between social media use and physical activity behavior.

Path	Effect	Effect value	S. E.	95% C. I.
H2: SMU → BRI → PAB	Total effect	0.237	0.058	[0.120, 0.348]
	Direct effect	0.179	0.065	[0.052, 0.305]
	Indirect effect	0.057	0.021	[0.018, 0.102]
H3: SMU → BON → PAB	Total effect	0.294	0.060	[0.178, 0.412]
	Direct effect	0.179	0.065	[0.052, 0.305]
	Indirect effect	0.114	0.030	[0.058,0.178]
H4: SMU → STR → PAB	Total effect	0.254	0.059	[0.139, 0.370]
	Direct effect	0.179	0.065	[0.052, 0.305]
	Indirect effect	0.074	0.027	[0.025, 0.130]
H5: SMU → SE → PAB	Total effect	0.242	0.065	[0.114, 0.371]
	Direct effect	0.179	0.065	[0.052, 0.305]
	Indirect effect	0.063	0.020	[0.114, 0.371]
H6: SMU→ STR → SE → PAB	Total effect	0.359	0.056	[0.247, 0.470]
	Direct effect	0.179	0.065	[0.052, 0.305]
	Indirect effect	0.180	0.031	[0.123, 0.243]
H7: SMU→ BON → SE → PAB	Total effect	0.715	0.106	[0.503, 0.922]
	Direct effect	0.179	0.065	[0.052, 0.305]
	Indirect effect	0.536	0.051	[0.430, 0.633]
H8: SMU → BRI → SE → PAB	Total effect	1.487	0.203	[1.086, 1.886]
	Direct effect	0.179	0.065	[0.052, 0.305]
	Indirect effect	1.308	0.203	[1.021, 1.586]

In addition, a chain mediation effect was identified: social media use indirectly influences physical activity behavior through the pathway, SMU → social capital (structural, bonding, and bridging social capital) → SE → PAB. Structural social capital (total effect = 0.359, indirect effect = 0.180), bonding social capital (total effect = 0.715, indirect effect = 0.536), and bridging social capital (total effect = 1.487, indirect effect = 1.308) all exhibit mediation effects with confidence intervals that exclude zero (at the 95% confidence level). Since all Bootstrap confidence intervals exclude zero, Hypotheses H6, H7, and H8 are fully supported.

## Interpretation and implications of findings

This study investigates how social media use influences physical activity behaviors in older adults. In contrast to the study by Schlenk et al. (2021), which focused on the elderly population in the U.S., this research, based on a survey in Shandong Province, China, finds a significant positive direct relationship between social media use and physical activity behavior among older adults. Despite this, the findings are consistent with several earlier studies (Jiang et al., 2024), which highlight the mediating role of factors such as structural social capital, bonding social capital, bridging social capital, and self-efficacy in the relationship between social media use and physical activity in older adults. This research expands on these findings by examining how social media use enhances physical activity behaviors through these mediating mechanisms. Building on prior studies, the results show that social media use positively influences physical activity through various mediators, including structural social capital, bonding social capital, bridging social capital, and self-efficacy.

In line with previous research on younger adults (Rutter et al., 2021; Durau et al., 2022), older adults who actively engage in social media for exercise are more likely to participate in physical activities. Specifically, various dimensions of social capital—such as structural social capital, bonding social capital, bridging social capital, and self-efficacy—act as important mediators in the relationship between social media usage and physical activity. This study highlights the essential role of social media in promoting physical activity among older adults, fostering social capital, and contributing to successful aging, thus adding to the growing body of literature in social media and communication studies. The results suggest that social media use can help older adults boost their physical activity levels and increase their self-efficacy by acquiring social capital, encouraging further physical activity. Given the risk factors older adults face—such as physical limitations, functional decline, and muscle loss—they are more likely to experience a decline in physical abilities. Therefore, social media helps older adults establish physical activity habits and offers more opportunities for physical exercise, effectively improving their levels of physical activity.

Notably, the mediating effect of bridging social capital, self-efficacy, and physical activity was substantially larger than the other indirect paths. This result suggests that bridging social capital, which represents loose ties with diverse social groups and access to non-redundant resources, may be particularly influential in promoting physical activity among older adults. Prior studies have suggested that such heterogeneous networks can provide broader informational support, favorable social comparisons, and new behavioral norms, all conducive to enhancing self-efficacy (Han and Zhai, 2024). However, the large effect size (1.308) may also indicate potential model sensitivity or overestimation related to measurement structure, especially in cross-sectional SEM.

### Theoretical contributions

This study makes several significant theoretical contributions that are worth highlighting. First, while research on social media usage and its impact on physical activity has expanded over the past decade, studies examining the relationship between social media use and physical activity behavior in older adults remain relatively scarce. By focusing on the elderly population, this study explores how social media usage influences physical activity behavior, offering a fresh perspective. Furthermore, the study makes notable progress in the literature on healthy aging, particularly regarding how social media affects physical activity through various dimensions of social capital (structural, bonding, and bridging social capital) and self-efficacy as mediating factors. These elements have been shown to mediate physical activity behavior in older adults significantly. Specifically, the social connections fostered by social media and the enhancement of self-efficacy are key drivers of physical activity among older adults. The results provide preliminary support for this model, helping to explain variations in physical activity behavior among the elderly. However, this study offers only preliminary evidence regarding the role of social media in influencing older adults’ physical activity, leaving several important questions to be explored further. For instance, older adults generally have less exposure to information and communication technology (ICT) and may lack the skills to use new technologies effectively, which worsens the digital divide. Thus, the research assumes that older adults can use social media equally without addressing factors like technological competence and self-efficacy that may impact the effectiveness of social media usage. Moreover, this study demonstrates the positive impact of social media on physical activity in older adults and highlights the chain-mediating effect of self-efficacy. This finding prompts further reflection on how individual differences may moderate the relationship between social media use and physical activity in older adults. For example, we discovered that older adults who are more comfortable with self-disclosure on social media experience a significant boost in self-efficacy when using WeChat. This mediating role of self-efficacy adds depth to the existing literature on how social media influences physical activity behavior differences among older adults.

### Practical implications

This study has significant practical implications for both older adults and policymakers. The results indicate that social media use aids older adults in building social capital, which closely correlates with increased self-efficacy and greater physical activity. Social media provides a unique advantage in encouraging older adults to engage in physical activity, particularly for less active ones. Through social media, they can partake in physical activities with close family members and friends and establish new social connections with acquaintances or even strangers. Therefore, the government should continue to invest in enhancing ICT infrastructure, reducing broadband internet costs, and ensuring that older adults have equal internet and social media access, thereby increasing their usage. Additionally, when creating health promotion strategies for aging populations, policymakers should consider regional and educational disparities and design more tailored interventions. For instance, local community centers could motivate older adults to engage in physical activities by organizing a variety of options, such as dance, chess, hiking, and traditional martial arts, to help improve their fitness and overall health.

### Limitations and future research directions

The findings of this study should be interpreted in light of several limitations. First, while the study focuses on social media and its direct and indirect effects on physical activity behavior in older adults, it may overlook how other patterns of social media use could influence physical activity. For instance, some older adults may primarily use social media for entertainment, such as watching live streams or political participation, which could affect physical activity differently. Therefore, future research should investigate how diverse social media usage patterns affect physical activity in older adults. Second, this study measures social media use using five specific items designed to capture various aspects of social media use. However, it does not consider certain important activities, such as watching fitness live streams on platforms like WeChat or Douyin and interacting with others. Future studies could expand the measurement of social media use to include a broader range of social media behaviors. To gain a deeper understanding of how different usage dimensions affect physical activity, researchers could analyze each aspect of social media use separately, particularly looking at how activities such as watching fitness streams and other forms of interaction impact older adults’ overall use of social media. Third, this study uses cross-sectional data, which limits its ability to establish causal relationships. While we found a correlation between social media use and physical activity, we cannot determine the direction of causality. Future research should utilize longitudinal data or experimental designs to establish causal inferences better. Lastly, while this study shows a positive correlation between social media use and physical activity in older adults and examines the mediating roles of social capital and self-efficacy, it is unclear whether this framework applies to other economically developed regions or countries. Future research could include more diverse samples, such as stratified sampling, to include older adults with limited exposure to or familiarity with digital technologies to test this model’s generalizability further.

## Conclusion

With the advancement of ICT and the widespread use of smartphones, social media has become a key tool for promoting home fitness, physical activity, and overall health. While research on the effects of social media use on physical health has increased in recent years, studies examining the link between social media use and physical activity behavior in older adults remain relatively scarce. This study’s findings demonstrate a direct relationship between social media use and physical activity behavior in older adults. The research further identifies that social capital (including structural, bonding, and bridging social capital) and self-efficacy play important sequential mediating roles in the relationship between social media usage and physical activity behavior in the elderly. These results confirm the positive correlation between social media use and physical activity observed in previous studies. Additionally, the study highlights that understanding the patterns of social media use and its application in promoting physical activity can help older adults overcome the physical decline and health challenges that come with aging, thereby improving their overall health and quality of life.

## Data Availability

The raw data supporting the conclusions of this article will be made available by the authors, without undue reservation.
